# Identification of epipolythiodioxopiperazines HDN-1 and chaetocin as novel inhibitor of heat shock protein 90

**DOI:** 10.18632/oncotarget.3029

**Published:** 2015-01-24

**Authors:** Xiaoping Song, Zhimin Zhao, Xin Qi, Shuai Tang, Qiang Wang, Tianjiao Zhu, Qianqun Gu, Ming Liu, Jing Li

**Affiliations:** ^1^ Key Laboratory of Marine Drugs, Ministry of Education, School of Medicine and Pharmacy, Ocean University of China, Qingdao, P. R. China; ^2^ Division of Antitumor Pharmacology, State Key Laboratory of Drug Research, Shanghai Institute of Materia Medica, Chinese Academy of Sciences, Shanghai, P. R. China; ^3^ Department of Pharmacy, School of Pharmaceutical Sciences, South-Central University for Nationalities, Wuhan, P. R. China

**Keywords:** pipolythiodioxopiperazines, HDN-1, chaetocin, inhibitor, Hsp90

## Abstract

The molecular chaperone heat shock protein 90 (Hsp90) has emerged as an important target for cancer treatment. HDN-1, an epipolythiopiperazine-2, 5-diones (ETPs) compound, was here identified as a new Hsp90 inhibitor. HDN-1 bound directly to C-terminus of Hsp90α, resulting in a potential conformational change that interfered with the binding of 17-AAG and novobiocin to Hsp90α. In contrast, association of 17-AAG, novobiocin or ATP with Hsp90α did not prevent the binding HDN-1 to Hsp90α. HDN-1 in combination with 17-AAG exhibited an enhanced inhibitory effect on non-small lung cancer cell proliferation. Molecular docking analyses revealed that HDN-1 bound to Hsp90α at C-terminal 526–570 region. In addition, HDN-1 degraded multiple oncoproteins and promoted EGF-induced wild type and mutated EGFR downregulation. Notably, chaetocin, used as a SUV39H1 inhibitor with similar structure to HDN-1, bound to Hsp90 and degraded Hsp90 client proteins and SUV39H1 as did HDN-1. These results indicate that HDN-1 and chaetocin are inhibitors of Hsp90 and that SUV39H1 is a novel client protein of Hsp90.

## INTRODUCTION

Heat shock protein 90 (Hsp90) is an evolutionarily conserved molecular chaperone. Human Hsp90 has four human isoforms. The two cytosolic isoforms in mammalian cells include the major, inducible Hsp90α and the minor, constitutively expressed, Hsp90β [1]. Hsp90 participates in stabilizing and activating more than 200 proteins, referred to as Hsp90 ‘clients’. Many of Hsp90 client proteins are essential for cell signaling and adaptive responses to stress [2, 3]. Importantly, some Hsp90 clients are *bona fide* oncoproteins, linked to all six hallmarks of cancer as defined by Hanahan and Weinberg, and inhibitor of Hsp90 was seemed to be able to simultaneously affect all six hallmarks of cancer [4]. Hsp90 is frequently upregulated in many solid tumors and hematological malignancies, protecting an array of mutated and overexpressed oncoproteins from misfolding and degradation and activating them. These oncoproteins include EGFR, Akt, cyclinD1, BCR-ABL, ERB-B2, CRAF, BRAF, MET, VEGFR, FLT3, androgen and estrogen receptors, and hypoxia-inducible factor (HIF)-1α [5, 6]. Inhibition of Hsp90 induces apoptosis through inhibition of the multiple growth signalings [7], and Hsp90 has been recognized as a crucial facilitator of oncogene addiction and cancer cell survival and has emerged as an important target in cancer therapeutics [8, 9].

Hsp90 forms a homodimer and each monomer contains three flexibly linked regions, an N-terminal domain (1–275 aa), middle domain (275–444 aa), and a C-terminal domain (444–677 aa) [10, 11]. N-terminal domain binds to ATP, co-chaperones, and potentially client proteins. Middle domain, which contains a catalytic arginine required for the ATPase activity, binds to co-chaperones and is thought to be the major client-protein binding domain. C-terminal domain contains a second ATP-binding site and the major dimerization interface, which makes Hsp90 a constitutive dimer. The C-terminus is a highly conserved MEEVD motif, which binds to TPR-containing co-chaperones [2]. Early attempts of drug development concentrated on blocking ATP binding at the N-terminal domain of Hsp90. Two natural products, geldanamycin (GA) and radiciol, and other synthetic small-molecule inhibitors, such as 17-AAG, IPI-504, KF58333, AUY922A, BIIB021, and SNX2112, have been shown to possess anti-proliferative activity and target the ATP-binding site in the N-terminal domain of Hsp90. Up to now, 13 Hsp90 inhibitors representing multiple drug classes are undergoing clinical evaluation, and many more compounds are in pre-clinical development [9]. However, human clinical trials involving these Hsp90 N-terminal inhibitors revealed that most of these inhibitors exhibit unfavorable toxicity profiles and tendency to induce expression of cytoprotective Hsp70 proteins [5, 12]. Because of the growing understanding of the mechanisms underlying the function of Hsp90 in malignant transformation, C-terminal/middle domains of Hsp90 inhibitor, co-chaperone/Hsp90 interactions inhibitors, client/Hsp90 associations, and cell surface Hsp90 inhibitors have now been under investigation [13, 14].

**Figure 1:**
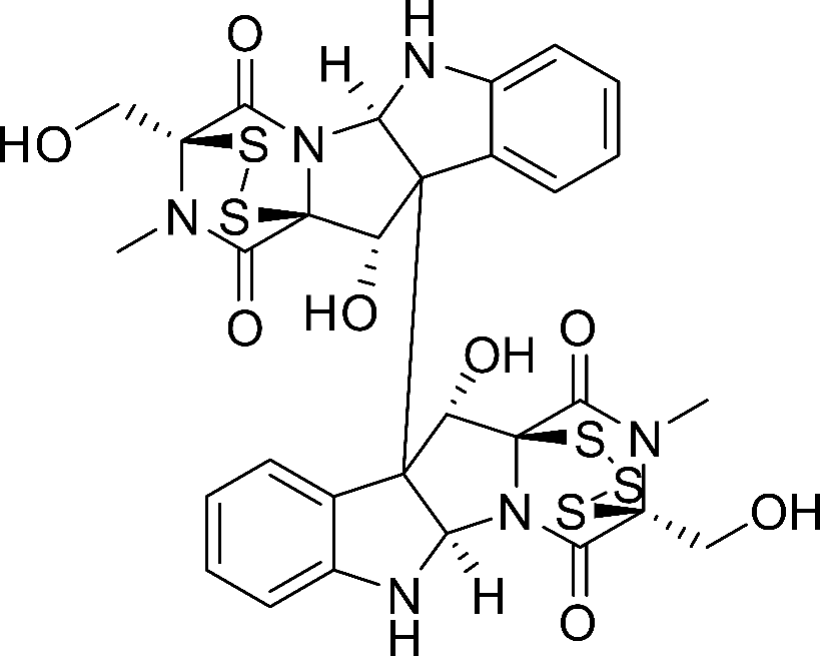
Chemical structure of HDN-1

Epipolythiopiperazine-2, 5-diones (ETPs) constitute an important class of biologically active compounds, characterized by a bridged polysulfide piperazine ring. HDN-1 (Figure 1) is a novel ETPs obtained from the antarctic fungus *Oidiodendron truncatum* GW3–13, which was isolated from the soil under lichens near to the Great Wall station (Chinese Antarctic station). HDN-1 has significant cytotoxic activities against various human cancer cell lines [15]. Our preliminary studies revealed that HDN-1 simultaneously inhibited various proteins expression, which suggested that HDN-1 is a new Hsp90 inhibitor. In the present study, we investigated the relationship between HDN-1 and Hsp90, and examined the effect of HDN-1 on Hsp90 regulation compared with that exhibited by the N-terminal inhibitor 17-AAG and C-terminal inhibitor novobiocin. Our results demonstrated that HDN-1 is a novel C-terminal inhibitor of Hsp90. In addition, we revealed that chaetocin functions as inhibitor of Hsp90 and SUV39H1 is a new client protein of Hsp90.

## RESULTS

### HDN-1 binds to Hsp90α

**Figure 2:**
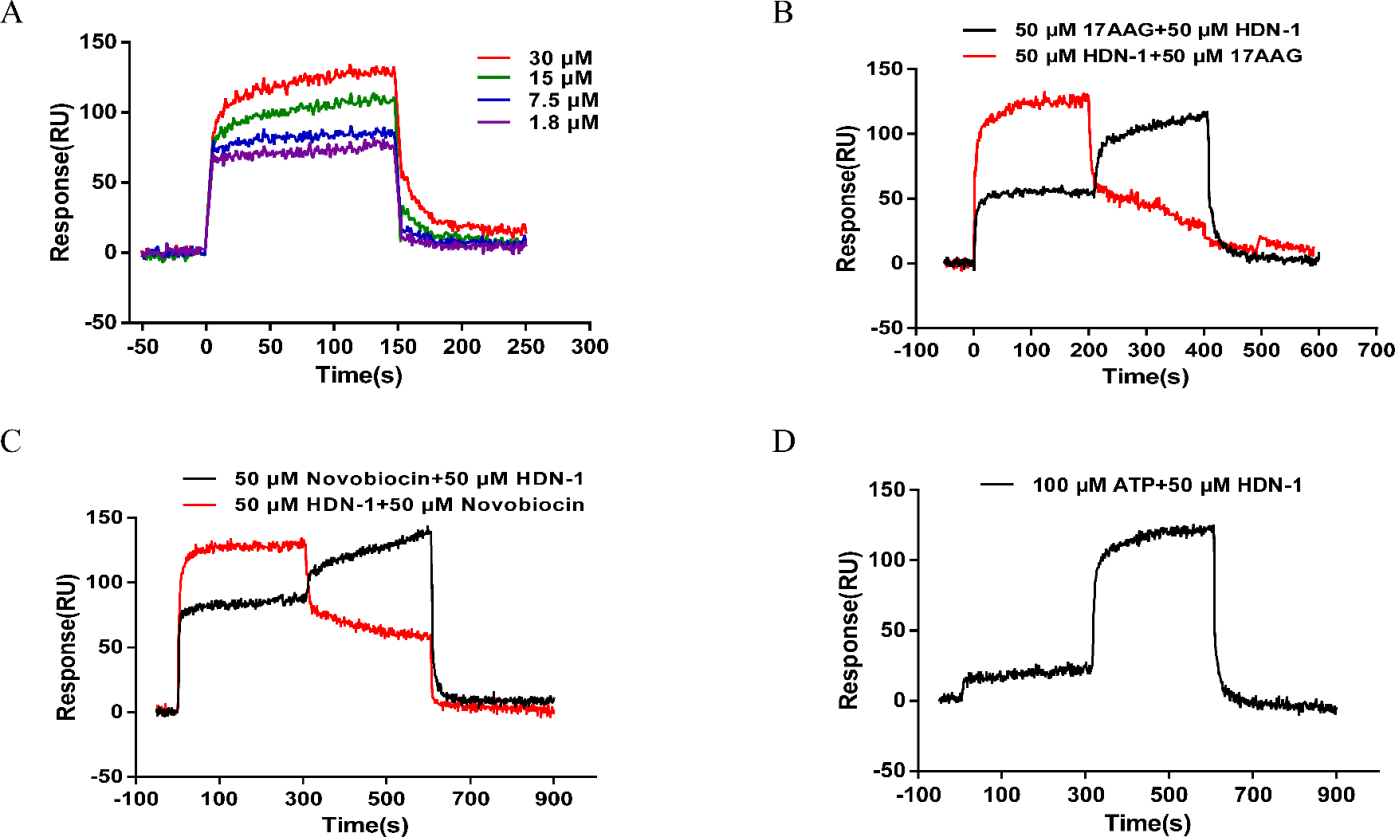
Interaction of HDN-1 with Hsp90 immobilized on a GLH sensor chip in the absence and presence of 17-AAG, novobiocin, and ATP The binding RU was recorded as binding potency. **(A)** HDN-1 binding to full length Hsp90α. Various concentrations (1.8–30 μM) of HDN-1 were injected into Hsp90α-immobilized chip, and RU values were recorded. **(B)** The interplay of 17-AAG (50 μM) and HDN-1 (50 μM) on binding to Hsp90α. Black line: 50 μM 17-AAG was injected onto Hsp90α-immobilized chip and the RU values were recorded in succession. 50 μM 17-AAG was replaced by 50 μM HDN-1 200 seconds after the injection, and RU values were recorded unceasingly. Red line: 50 μM HDN-1 was first injected onto Hsp90α-immobilized chip and was replaced by 50 μM 17-AAG 200 seconds after the first injection. **(C)** The interplay of novobocin and HDN-1 on binding to Hsp90α. Black line: 50 μM novobocin was injected onto Hsp90α-immobilized chip and RU values were recorded in succession. novobocin was replaced by 50 μM HDN-1 300 seconds after novobocin injection, and RU values were recorded; Red line: 50 μM HDN-1 was first injected into Hsp90α-immobilized chip and was replaced by 50 μM novobocin 300 seconds after HDN-1 injection. **(D)** The ATP did not affect the HDN-1's binding to Hsp90α. 100 μM ATP was injected into Hsp90α-immobilized chip, and RU values were recorded, and was replaced by 50 μM HDN-1300 seconds after ATP injection.

To investigate whether HDN-1 directly binds to Hsp90α, we used surface plasmon resonance (SPR) to determine the interaction between HDN-1 and Hsp90α, which was biotinylated and immobilized onto a streptavidin-coated sensor chip. As shown in Figure 2A, typical sensorgrams of the interaction between HDN-1 and Hsp90α were obtained at 30, 15, 7.5 and 1.8 μM of HDN-1. The dissociation constant (Kd values) of HDN-1 was 14.6 μM, indicating that HDN-1 binds to Hsp90α with moderate affinity. To identify the binding site of HDN-1, we injected 17-AAG, novobiocin or ATP over the chip before or after HDN-1 inclusion. We found that HDN-1 was able to associate with Hsp90α that was pre-bounded with 17-AAG (Figure 2B), novobiocin (Figure 2C) or ATP (Figure 2D). In contrast, a pre-association of HDN-1 with Hsp90α reduced the binding of Hsp90α to novobiocin (Figure 2C) or ATP (Figure 2D). These results strongly suggest that HDN-1 binds to Hsp90α in a manner different from 17-AAG, novobiocin and ATP. The binding HDN-1 to Hsp90α may alter Hsp90α conformation that prevents the access of Hsp90α to 17-AAG, novobiocin and ATP.

### HDN-1 binds to C-terminus of Hsp90α

**Figure 3:**
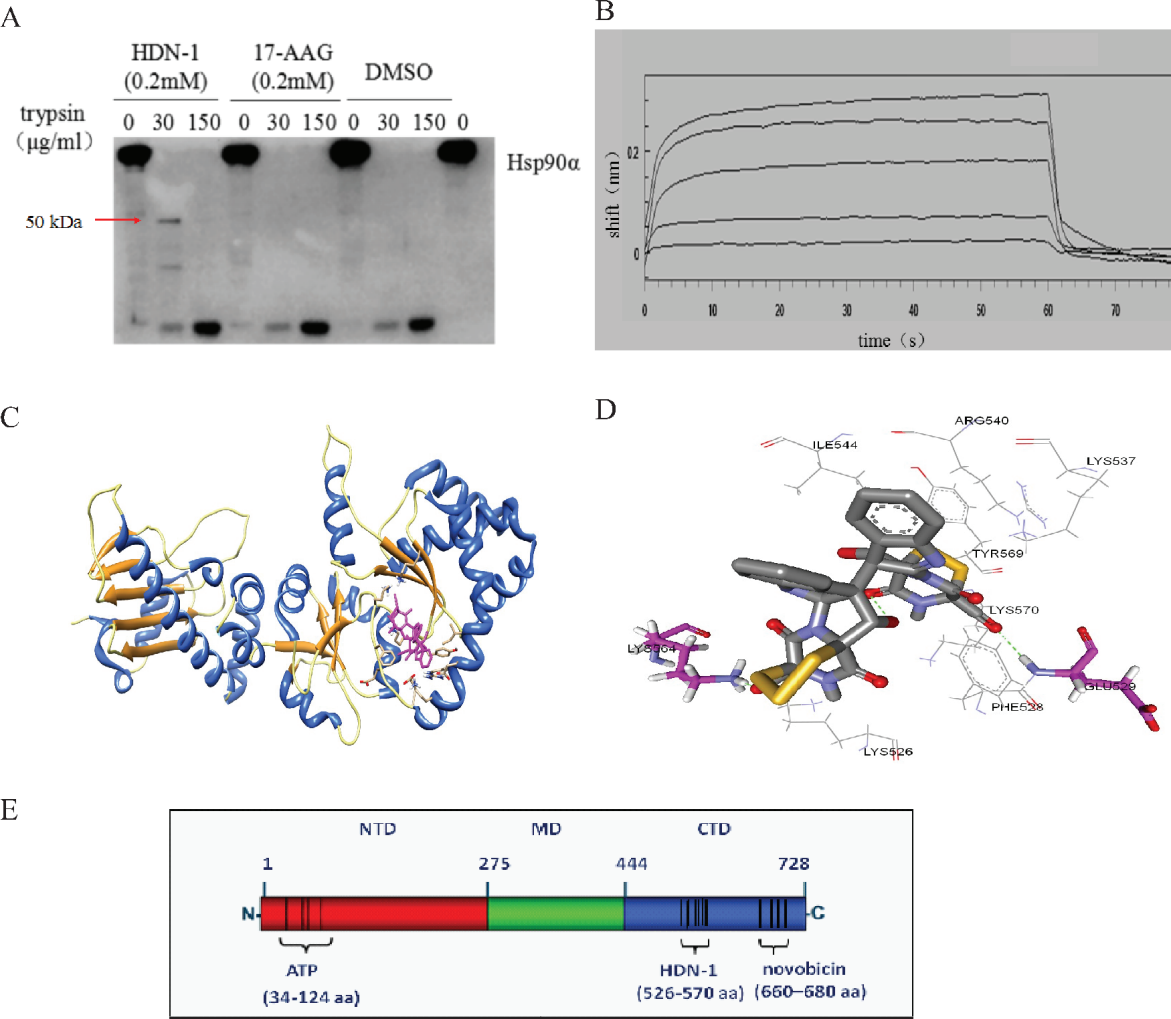
HDN-1 binds to C-terminus of Hsp90α **(A)** Proteolytic fingerprinting of HDN-1 and 17-AAG on Hsp90α. 0.6 μg Hsp90α was incubated with 200 μM HDN-1 or 200 μM 17-AAG, then digested by TPCK-treated trypsin and analyzed by Western blotting with an anti-Hsp90α (C-terminus) antibody. **(B)** HDN-1 binds to C-terminal Hsp90α. The binding interactions were measured using the ForteBio's Octet system for the interaction between HDN-1 and biotin labeled C-Hsp90α that was immobilized on NHS-LCLC-biotin Superstreptavidin biosensors. The curve from top to bottom denotes 500, 250, 125, 62.5 and 31.25 μM of the concentration of HDN-1 respectively. **(C)** A schematic illustration of the interactions between HDN-1 and full length of Hsp90α. **(D)** An amplified view of HDN-1's binding to Hsp90α. **(E)** A figure illustrating the binding site of HDN-1 and various ligands to full-length Hsp90α.

To determine the region of Hsp90α that binds to HND-1, we used competitive inhibition assay based on fluorescence polarization. We found that N-terminal Hsp90α inhibitor 17-AAG competed with GA to bind to full-length Hsp90α (IC50; 0.298 μM) (Table 1). In contrast, HDN-1 did not affect GA's binding to Hsp90 even at 10 μM concentration. These results suggested that HDN-1 and 17-AAG bind to different regions of Hsp90α or the binding of HDN-1 to Hsp90α alters the Hsp90α structure that prevents the binding of 17-AAG. Given that the conformational changes of proteins could lead to different proteolytic fingerprintings [14], we performed proteolytic fingerprinting assay with purified full-length Hsp90α proteins. Hsp90α protein was incubated with 17-AAG or HDN-1 followed by limited trypsin digestion, the fragments containing the C-terminal domain of Hsp90α were detected by an antibody specifically recognizing epitopes within the C-terminus. As shown in Figure 3A, Hsp90α incubation with HDN-1 generated a 50 kDa C-terminal fragment. In contrast, this fragment was not detected after 17-AAG incubation. These results implicated that HDN-1 interacts with the C-terminal domain of Hsp90α and affects its degradation by trypsin.

**Table 1:** The effect of 17-AAG and HDN-1 on the binding of GA to Hsp90

Compounds	IC_50_(μM)
17-AAG	0.298
HDN-1	> 10

The combination of C-terminal and N-terminal Hsp90 inhibitor may induce enhancing effects [16, 17]. We next examined the effects of 17-AAG (100, 200, 300 nM) and HDN-1 (100, 200 nM), either alone or in combination, on growth inhibition of H1975 non-small cell lung cancer cells by the MTT assay (The Q value was calculated according to Jin's formula [18]). As shown in Table 2, co-treatment with 17-AAG and HDN-1 greatly enhanced the cell proliferation inhibition in a dosage-dependent manner compared to the treatment with 17-AAG or HDN-1 alone (Q value were greater than 0.85), suggesting that HDN-1 binds to C-terminus of Hsp90. This finding was further supported by SPR spectroscopy with recombinant C-terminal Hsp90 fragment (535–732aa) of human Hsp90α immobilized onto chip. Figure 3B shows that HDN-1 was able to bind to C-terminus of Hsp90α with the Kd value of 201 μM.

**Table 2:** Effects of HDN-1 and 17-AAG on H1975 cell proliferation

		Inhibition Rate		
17-AAG/nM	HDN-1/nM	Mean	SD	Q (value)
0	0	0.000	0.037	
0	100	0.051	0.061	
0	200	0.371	0.041	
100	0	0.070	0.028	
200	0	0.267	0.028	
300	0	0.393	0.017	
100	100	0.124	0.039	1.060
200	100	0.350	0.049	1.150
300	100	0.431	0.016	1.017
100	200	0.399	0.026	1.061
200	200	0.704	0.019	1.306
300	200	0.704	0.012	1.139

To predict the potential binding site of HDN-1, we performed molecular docking analyses. As shown in Figure 3C and 3D, HDN-1 was able to dock into C-terminus of Hsp90α. The hydroxyl groups in the HDN-1 molecule formed two hydrogen bonds (< 3.0 Å) with residues Glu529 and Lys564, and the rest of the molecule were stabilized by hydrophobic interactions with Lys526, Phe528, Lys537, Arg540, Ile544, Tyr569, and Lys570. These results suggest that HDN-1 binds to a novel site different from the reported bind sites of Hsp90α N-terminal and C-terminal inhibitors [14], and this novel binding site is probably at 526–570 region of Hsp90α (Figure 3E).

### HDN-1 induces degradation of multiple oncoproteins

**Figure 4:**
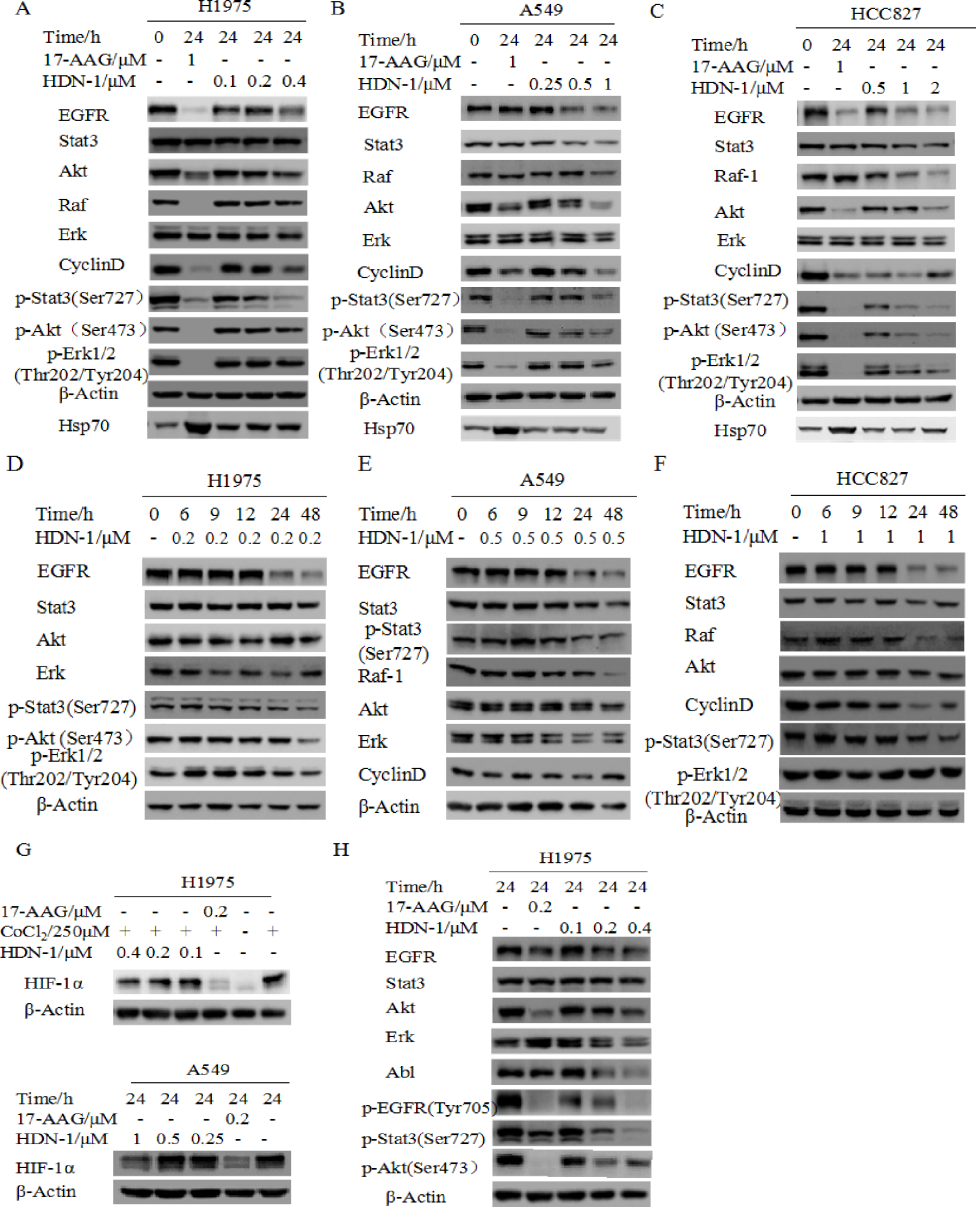
HDN-1 induces degradation of multiple oncoproteins HDN-1 treatment induced degradation of Hsp90 client proteins in H1975 cells **(A, D)**, A549 cells **(B, E)** and HCC827 **(C, F)** in dosage- and time-dependent manners. Cells were incubated with HDN-1 at indicated concentrations and time, and then subjected to Western blotting assay with indicated antibodies. **(G)** HDN-1 induced degradation of HIF-1α in H1975 and A549 cells under hypoxic condition. Cells were incubated with HDN-1 at indicated concentrations in the presence of 250 μM CoCl_2_ or cultured in low oxygen condition (5% CO_2_ and 2% O_2_). **(H)** The sensitivity of different Hsp90 client proteins to HDN-1 or 17-AAG treatment in H1975 cells. Cells were incubated with HDN-1 or 17-AAG at indicated concentrations, and then analyzed by Western blotting.

We first tested the growth inhibitory effect of HDN-1 on three non-small cell lung cancer cell lines H1975, HCC827 and A549. HDN-1 inhibited the proliferation of these cancer cell lines with an IC_50_ of 0.22, 0.54, and 1.06 μM, respectively (Table 3). We showed that H1975 cells, which contain T790M mutation were resistant to conventional EGFR inhibitors, were sensitive to HDN-1 treatment. The Hsp90 chaperone complex regulates many client oncoproteins that play key roles in tumor formation and progression [3, 4, 5]. Therefore, we next examined whether HDN-1 induced degradation of Hsp90 client oncoproteins. We found that HDN-1 treatment reduced the expression levels of EGFR, Stat3, Akt, and Erk, and their active phosphorylated forms and downregulated the expression of Raf and Cyclin D1 in H1975, HCC827, and A549 cells, in dosage- (Figure 4A–4C) and time- (Figure 4D–4F) dependent manners. These results suggested that binding of HDN-1 to Hsp90 resulted in inhibition of Hsp90 activity and induced degradation of many client oncoproteins. Intriguingly, we noticed that HDN-1 did not obviously affect Hsp70 expression as did 17-AAG (Figure 4A–4C). It is reported that increase of Hsp70 is a striking feature induced by Hsp90 N-terminal inhibitors, such as 17-AAG [12], while Hsp90 C-terminal inhibitors do not regulate Hsp70 expression [19]. Thus, our results are consistent with the previous reports and further support that HDN-1 is an Hsp90 C-terminal inhibitor.

**Table 3:** IC_50_s of HDN-1 for non-small cell lung cancer cell proliferation

Cell lines	IC_50_(μM)
H1975	0.22
A549	0.54
HCC827	1.06

Solid tumors typically exhibit regions of poor oxygen supply or hypoxia, and the HIF-1α regulates an adaptive response to hypoxia. HIF-1α expression has been correlated with resistance to radiation and chemotherapy as well as poor clinical prognosis. Since HIF-1α is a client protein of Hsp90 [20], we speculated that HIF-1α expression is regulated by HDN-1. As expected, HIF-1α was degraded after cells were exposed to HDN-1 under CoCl_2_-induced hypoxia (Figure 4G, top panel) or under 5% CO_2_ and 2% O_2_ condition (Figure 4G, bottom panel).

Hsp90 client proteins may response to Hsp90 inhibitors differently. We next compared the sensitivity of different client proteins to HDN-1 and 17-AAG. We observed that some client proteins including Abl, p-Stat3, and Erk were more sensitive to HDN-1 than 17-AAG, while others, such as EGFR and Akt, were more susceptible to 17-AAG than to HDN-1 (Figure 4H). One possible explanation is that HDN-1 is an Hsp90 C-terminal inhibitor and it may have a different selectivity of client proteins compared with N-terminal inhibitor 17-AAG.

### HDN-1 promotes ligand-induced EGFR down-regulation

**Figure 5:**
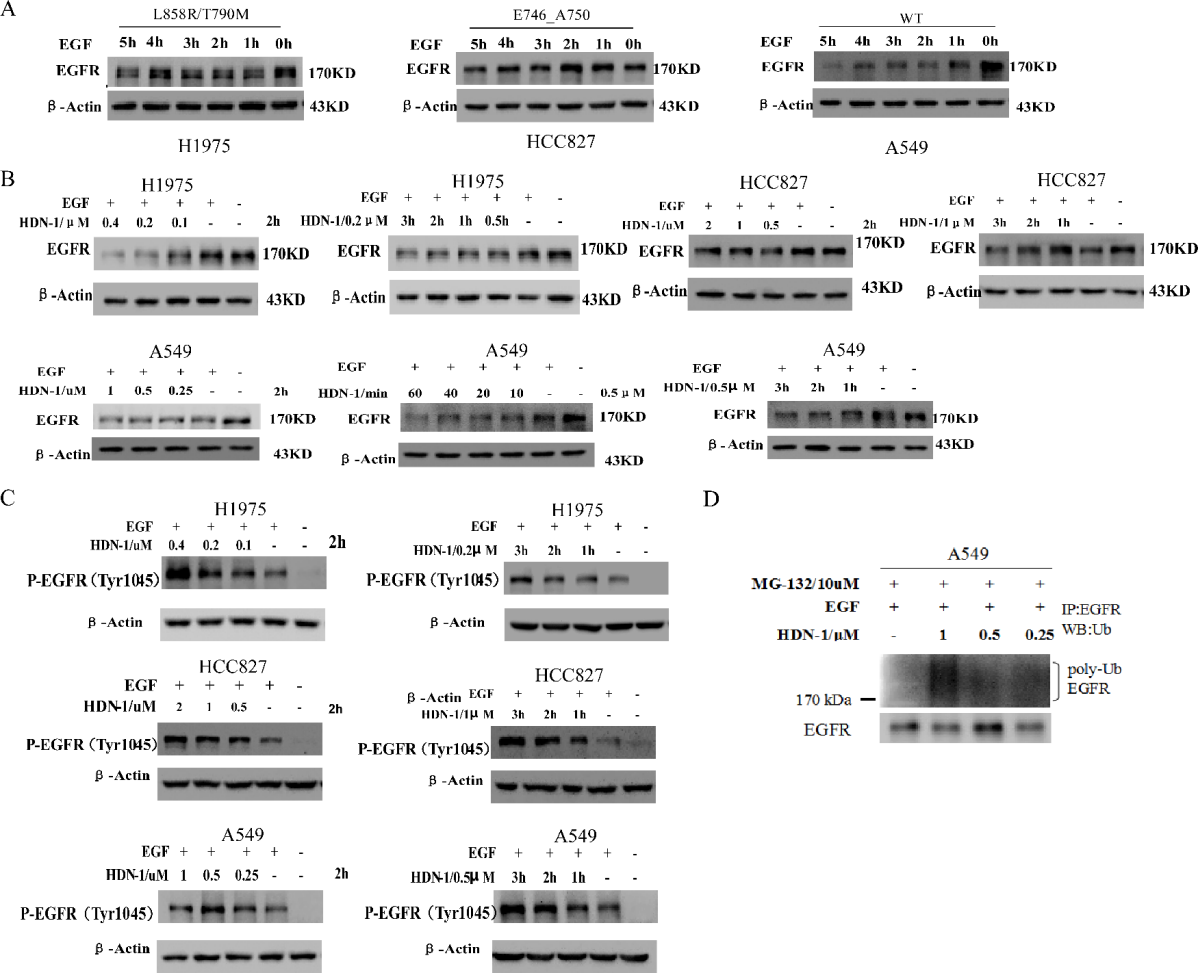
HDN-1 promotes EGF-induced EGFR downregulation **(A)** EGFR mutants in H1975 and HCC827 cells were resistant to be degraded upon EGF treatment. A549, H1975 or HCC827 cells were cultured overnight with serum free medium and incubated with 25 ng/ml EGF at indicated time. EGFR expression was determined by Western blotting assay. **(B, C)** HDN-1 promoted the reduction of EGFR expression and upregulation of EGFR Tyr1045 phosphorylation upon EGF stimulation in A549, H1975 and HCC827 cells. A549, H1975 or HCC827 cells were incubated with HDN-1 at indicated concentrations and time in the presence of 25 ng/ml EGF. EGFR and EGFR pY1045 were determined by Western blotting assay. **(D)** HDN-1 increased the level of EGFR ubiquitination in A549 cell. A549 cells were pretreated with 10 μM MG132 for 1 h, and then incubated with HDN-1 at indicated concentrations in the presence with 25 ng/ml EGF for 30 min. The levels of EGFR ubiquitination were detected by an antibody for ubiquitin.

In response to ligand stimulation, EGFR undergoes a process of desensitization involving endocytosis, ubiquitination, and lysosomal degradation, which is called ligand-induced receptor downregulation (LIRD). However, LIRD was impaired in EGFR mutant-expressing cells. It is reported that Hsp90 impaired Cbl-mediated receptor downregulation, and inhibition of Hsp90 function restores LIRD [21]. To further evaluate the effect of HDN-1 on EGFR degradation, we examined EGFR levels in the cells treated with EGF for 1 to 5 h. In A549 cells with wild-type EGFR, EGFR level was markedly reduced after EGF stimulation for 1 h. In contrast, H1975 and HCC827 cells with EGFR mutant maintained EGFR levels upon EGF stimulation (Figure 5A). Of interest, HDN-1 treatment largely reduces the EGFR levels in EGF-treated A549, H1975 and HCC827 cells in dosage- and time-dependent manners (Figure 5B). This suggested that HDN-1 facilitated EGF-induced EGFR downregulation independent of the EGFR mutation status.

Phosphorylation of EGFR at Tyr1045 creates a major docking site for the adaptor protein c-Cbl, leading to receptor ubiquitination and degradation following EGFR activation [22]. We found that HDN-1 upregulated the phosphorylation of EGFR at Tyr1045 in the three types of lung cancer cells in dosage- and time-dependent manners (Figure 5C). In addition, HDN-1 increased the level of EGFR ubiquitination in A549 cells (Figure 5D). All these results suggested that HDN-1 promotes EGFR endocytosis and degradation through inhibition of Hsp90.

### Chaetocin binds to and inhibits Hsp90

**Figure 6:**
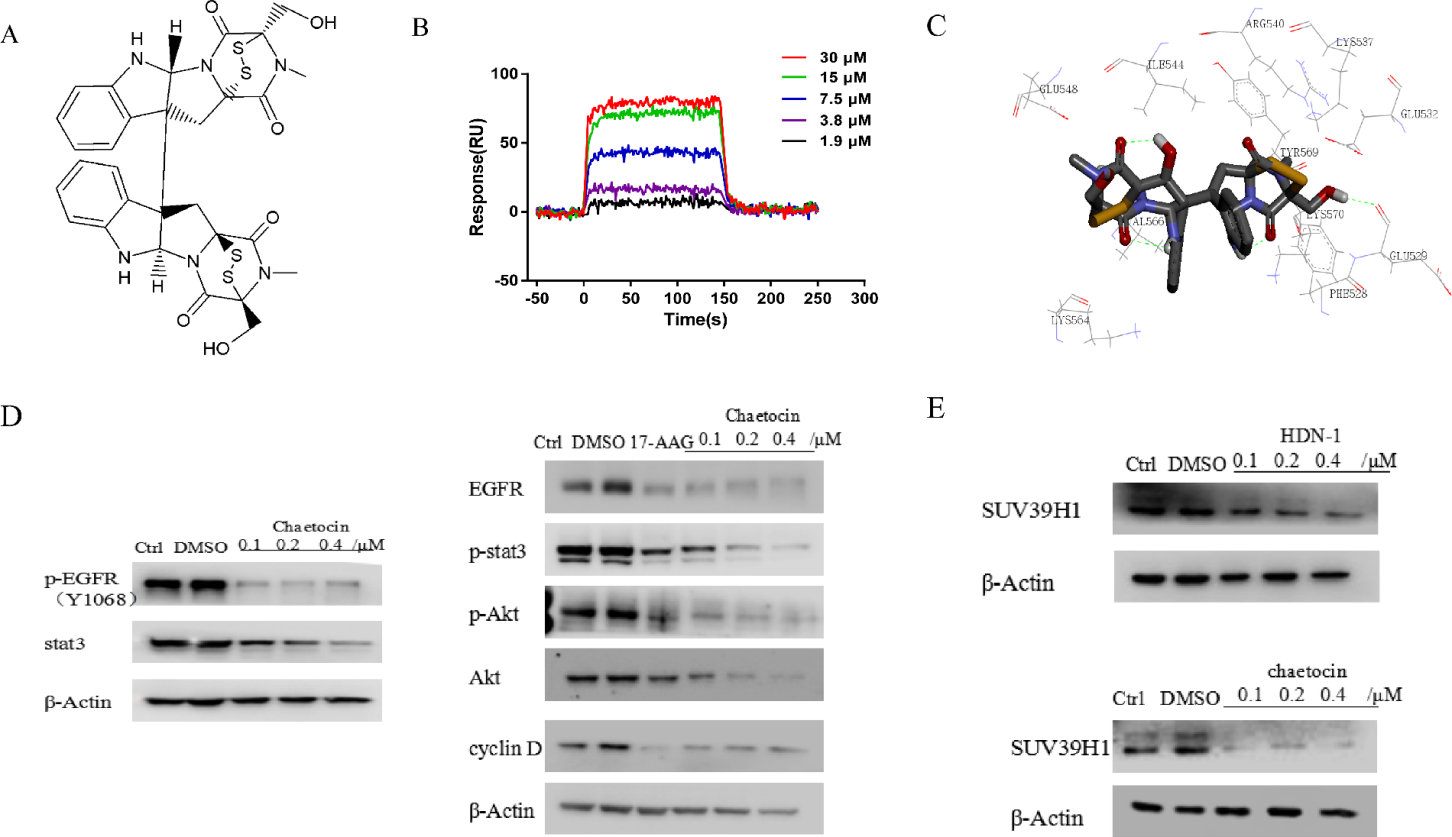
Chaetocin binds to and inhibits Hsp90 **(A)** Chemical structure of chaetocin. **(B)** Interaction of chaetocin with Hsp90α. Various concentrations of chaetocin were injected onto Hsp90α-immobilized chip, and RU values were recorded. **(C)** A schematic illustration of the interaction between chaetocin and full-length Hsp90α. **(D)** Chaetocin induced degradation of Hsp90 client proteins in H1975 cells. Cells were incubated with chaetocin at indicated concentrations for 24 h, then subjected to western blotting assay with indicated antibodies. **(E)** Both HDN-1 and chaetocin induced degradation of SUV39H1. Cells were incubated with HDN-1 or chaetocin at indicated concentrations for 24 h, then subjected to western blotting assay with antibody for SUV39H1.

HDN-1 is an analogue of chaetocin (Figure 6A), which is a fungal mycotoxin with histone methyltransferase SUV39H1 inhibitory activity [23]. To determine whether chaetocin also acts as an Hsp90 inhibitor, we determined chaetocin-Hsp90 binding and Hsp90 activity after chaetocin treatment. As shown in Figure 6B, chaetocin bound to Hsp90 with Kd values at 16.8 μM, which is close to the Kd values of HDN-1. The molecular docking analyses showed that chaetocin bound to Hsp90 with the same binding sites and interaction pattern as did HDN-1 (Figure 6C). Furthermore, chaetocin degraded client proteins, such as EGFR, p-EGFR, Akt, p-Akt, and Cyclin D1, at a dose similar to HDN-1 (Figure 6D). Notably, the level of SUV39H1 was found to decline upon chaetocin and HDN-1 treatment (Figure 6E). These results revealed that chaetocin is an inhibitor of Hsp90 and suggested that Hsp90 inhibition contribute to the inhibition on SUV39H1 and that SUV39H1 is a new client protein of Hsp90.

## DISCUSSION

Hsp90 has been intensively targeted for cancer treatment. Here, we identified HDN-1 and chaetocin as novel C-terminal Hsp90 inhibitors. Chaetocin was regarded as an inhibitor of SUV39H1 histone methyltransferase. Our results support that chaetocin has previously unknown function to bind to Hsp90 and to inhibit Hsp90 activity.

Hsp90α is reported to be essential to the survival of cancer cells, and the expression of Hsp90α is associated with different stress factors and leads to tumor progression, invasion and metastasis [24]. To investigate whether HDN-1 interacts with Hsp90α, we performed SPR analysis and showed that HDN-1 bound to the full-length and the C-terminus of Hsp90α with moderate affinity at Kd values of 14.6 and 201 μM, respectively. These affinities are higher than those of the current C-terminal inhibitors. Novobiocin, a C-terminal inhibitor used in cancer therapy, binds weakly to the C-terminus of ATP binding site (660–680 aa) of Hsp90α at approximately 3.3 mM [25, 26]. It is reported that 17-AAG binds to the ATP binding site (34–124 aa) of Hsp90 [27]. The binding of 17-AAG, novobiocin, and ATP to Hsp90α did not affect the association of HDN-1 with Hsp90α. However, HDN-1's binding to Hsp90α impairs the subsequent binding of 17-AAG and novobiocin, suggesting that HDN-1 induced conformational changes of Hsp90α that affects the access of other inhibitors. These findings were supported by proteolytic fingerprinting analyses showing that HDN-1 induced a distinct pattern of Hsp90 digestion to that induced by 17-AAG.

Csermely and his co-workers have reported that the C-terminal site becomes available for binding only after occupancy of the N-terminal site, and both the N- and C-terminal chaperone sites may contribute to Hsp90α function as chaperones [29]. Combination of novobiocin and GA had enhancing effects on proliferation inhibition and apoptosis induction of leukemia cells [30, 31]. In line with these reports, HDN-1 was found to have enhancing effect in combination with 17-AAG, an N-terminal Hsp90 inhibitor. The binding of HDN-1 to the C-terminal region of Hsp90α was further supported by molecular docking analyses, showing that 526–570 domain of Hsp90α with two hydrogen bonds binding at Glu529 and Lys564 is the interaction interface. It is reported that the Hsp90 C-terminus does not exhibit ATPase activity and is involved in the conformational rearrangement of Hsp90α upon ATP binding. The ATPase activity of Hsp90, which leads to a conformational change of the entire homodimer, is dependent upon the Hsp90 C-terminal region to trap the nucleotide during the ATPase cycle [32]. We found that ATP did not interfere HDN-1-Hsp90 binding measured by SPR spectroscopy while HDN-1 exhibited no effect on Hsp90α ATPase activity (data not shown). These results support that the binding of HDN-1 to Hsp90α is different from current C-terminal inhibitors, such as novobiocin, cisplatin, and epigallocatechin-3-gallate (EGCG), which bind to the Hsp90 C-terminal domain and interferes with nucleotide binding.

Activating mutations in the kinase domain of the EGFR in NSCLC are usually small exon 19 deletions and point mutations, and a replacement of leucine by arginine at codon 858 (L858R) in exon 21 is most commonly observed. Both types of mutations confer sensitivity to the anilinoquinazoline inhibitors of EGFR, gefitinib. Despite the initial responses of EGFR-mutant tumors to small-molecule tyrosine kinase inhibitors, resistance universally emerges over time. Acquired resistance has been associated with a second somatic mutation, resulting in a threonine-to-methionine substitution at position 790 (T790M) of EGFR. It have been reported that EGFR mutants depend on Hsp90 for stability. Crucial targets of Hsp90-active drugs vary in different cell types, and Hsp90 is permissive for the development of mutant EGFR-dependent lung cancers, these kinase domain mutants are degraded by Hsp90 inhibitors [20, 33]. Evaluation of the effects of HDN-1 on three NSCLC cell lines expressing wild type EGFR (A549), exon 19 deletions EGFR (HCC827), or T790M EGFR (H1975) showed that HDN-1 exhibited stronger inhibitory activity in the two cell lines with EGFR mutation than the cells with wild type EGFR. Importantly, HDN-1 degrades multiple client proteins, such as EGFR, Stat3, Akt, Erk, Raf, Cyclin D1, and HIF, all of which have been reported to be oncoproteins to promote cancer development. These results indicate that HDN-1 not only interacts with Hsp90, but also inhibit the cellular functions of Hsp90.

Inhibition of Hsp90 by targeting its N-terminus but not its C-terminus is often compensated by increased expression of Hsp70 as well as other heat shock proteins [12]. HDN-1, unlike 17-AAG, did not upregulate the expression of Hsp70, further supporting that HDN-1 is an Hsp90 C-terminal inhibitor. Furthermore, we found some client proteins including Abl, p-Stat3, Erk were more sensitive to HDN-1, while others, such as EGFR, Akt, p-Akt, p-Erk, were more sensitive to 17-AAG. These results suggest that inhibitors for the N-terminus and C-terminus have distinct effect on the selected degradation of Hsp90 client protein.

In normal tissues, the temporal and spatial distribution of extracellular growth factors determines the quantity and quality of intracellular signals. Once activated by stimulation, EGFR initiates both positive and negative processes that convey signals to downstream pathways via the interaction with adaptor proteins and remove receptors from the cell surface. In the latter case, the Cbl family of ubiquitin ligases plays a major role in ligand-dependent ubiquitination of many receptor tyrosine kinases [16]. EGFR activation results in the binding of Cbl to EGFR Tyr1045 for LIRD, and this EGFR downregulation was impaired in EGFR mutant-expressing cells. Hsp90 was detected in immunoprecipitates of EGFR and may contribute to the resistance of EGFR mutants to LIRD; hence, Hsp90 inhibitor would reverse the resistance [34]. Indeed, our studies showed that HDN-1 increased EGFR phosphorylation at Tyr1045 and promoted Cbl-mediated EGFR ubiquitination and LIRD. These results underscore the potential of HDN-1 to overcome the resistance of tumor cells to EGFR inhibitors.

HDN-1 is analogue of chaetocin, which was previously identified as a specific inhibitor of histone methyltransferase SUV39H1 [35]. However, another report shows that chaetocin was a nonspecific SUV39H1 inhibitor [36]. Here, we found that chaetocin inhibited multiple client proteins of Hsp90, which is unlikely due to the inhibition of SUV39H1. SUV39H1 specifically methylates K9 of histone H3 (H3K9me3) in the promoter regions of genes, leading to repression of genes and inhibition of cell cycle progression and cell proliferation. Downregulation of H3K9me3 has been observed in several types of human cancers (such as colorectal cancer, ovarian cancer, and lung cancer), which arise from either the deficiency of H3K9 methyltransferases or elevated activity or expression of H3K9 demethylases [37]. We demonstrated that chaetocin was inhibitors of Hsp90 and reduced SUV39H1 expression, suggesting that SUV39H1 is a new client protein of Hsp90 and chaetocin inhibits SUV39H1 by inhibiting Hsp90-mediated SUV39H1 stability.

In conclusion, we demonstrate HDN-1 is a novel inhibitor of Hsp90, binding to the 526–570 regions in the C-terminus, but not to the second ATP binding site. In addition, our results support that chaetocin is an inhibitor of Hsp90 and regulates SUV39H1 stability. As novel inhibitors of C-terminus of Hsp90, HDN-1 and chaetocin are promising anticancer compounds.

## METHODS

### Cell culture and reagents

The H1975 and HCC827 cell lines were purchased from ATCC and maintained in RPMI 1640 medium containing 10% FBS. The A549 cell line was maintained in F12K medium containing 10% FBS. HDN-1 was provided by School of Medicine and Pharmacy, Ocean University of China; Chaetocin, 17-AAG and TPCK-treated trypsin were purchased from Sigma-Aldrich; FITC-GA was purchased from Enzo Life Science. Hsp90α, C-Hsp90α, and EGF were from Abcam and Pepro Tech. Antibodies to detect EGFR, p-EGFR (Tyr1068), Akt, p-Akt, Erk, p-Erk, HIF-1α, p-EGFR (Tyr1045) and ubiquitination were obtained from Cell Signaling Technology. Antibodies to detect Stat3, p-Stat3, Raf, and CyclinD1 were purchased from Enzo Life Science. Primary antibody against SUV39H1 was purchased from Santa Cruz Biotechnology.

### Cell viability assay

A modified tetrazolium salt assay was used to measure the inhibition of cancer cell growth. Cancer cells were plated into a 96-well microtiter plate containing 0.1 ml of growth media/well for 24 h, then were incubated with HDN-1 at indicated concentrations dissolved in DMSO for 72 h. 3-(4,5-Dimethylthiazol-2-yl)-2,5- diphenyltetrazoliumbromide (MTT) was added to each well of a 96-well plate. After 4 h incubation, the formazan product was dissolved and quantitated spectrophotometrically at a wavelength of 560 nm. Percent viability of each sample was calculated from the A560 values as follows: % viability = (A560 nm sample / A560 nm DMSO-treated cells × 100). The IC_50_ was defined as the concentration that gave rise to 50% inhibition of cell viability.

### Western blotting and immunoprecipitation analysis

Cells were incubated with HDN-1 at indicated concentrations and time, then washed with PBS twice, disrupted on ice for 30 min in loading buffer, and boiled for 10 min. Protein concentration was determined with BCA reagent. Equal amounts of protein in cell lysates were separated by SDS-PAGE, transferred to membranes, immunoblotted with indicated primary and secondary antibodies, and detected by chemiluminescence with the enhanced chemiluminescence detection reagents (PIERCE). The antibodies of following proteins were used: EGFR, phosphorylated EGFR (Tyr1068), Akt, phosphorylated Akt (Ser473), MAPK, phosphorylated MAPK (Thr202/Tyr204), ubiquitin, Hsp70, β-Actin; Abl, Stat3, phosphorylated Stat3 (Ser727), Raf-1, cyclin D1. For immunoprecipitation assay, cell lysate protein was incubated overnight at 4°C with the designated antibody and protein G-Sepharose (Amersham Biosciences) was added overnight while rocking. Precipitates were washed three times with lysis buffer and once with PBS, resuspended in 2 × loading buffer, and resolved by SDS-PAGE followed by immunoblot analysis.

### Surface plasmon resonance spectroscopy (SPR)

SPR analysis was carried out with a ProteOn XPR36 instrument (Bio-Rad) or ForteBio's Octet RED96 to determine binding of various molecules to full length Hsp90α or C-terminal Hsp90α. For full length Hsp90α, Hsp90α was coupled to the surface of a GLH sensor chip using amine coupling. The coupling has been performed according to the manufacturer's instructions. Binding experiments were performed with multiple concentrations of HDN-1 and chaetocin running over the surface of the chip at 25°C, at a constant flow rate of 30 μl/min in PBS-T (10 mM Phosphate, 150 mM NaCl, pH 7.4 and 0.005% Tween20 [v/v]). The sensor chip surface was regenerated using 0.85% H_3_PO_4_. Changes in mass due to the binding response were recorded as resonance units (RU). To obtain the dissociation constant (Kd), these responses were fit to a 1:1 Langmuir binding model by nonlinear regression using the BiaEvaluation 4.1 software program. For interplay of HDN-1 with other molecules, 50 μM 17-AAG, 50 μM novobiocin, or 100 μM ATP was injected into Hsp90α-immobilized chip and the RU values were recorded in succession for indicated time, then these compounds were replaced by 50 μM HDN-1, and RU values were recorded unceasingly. Similar experiments were conversely performed and RU values were recorded. For C-terminal Hsp90α, the human purified C-Hsp90α was biotinylated using NHS-LCLC-biotin. Superstreptavidin biosensors were coated in a solution containing 10 μg/ml of biotinylated protein for 5 min at 30°C. A duplicate set of sensors was incubated in an assay buffer (1 × kinetics buffer of ForteBio's Inc.) with 1% dimethyl sulfoxide without protein for use as a background binding control. Both sets of sensors were blocked with a solution of 5 μM biocytin for 60 s at 30°C. Binding of samples (31.25–500 μM) to coated and uncoated reference sensors was measured over 60 s. Data analysis on the ForteBio's Octet RED96 instrument were performed using a double reference subtraction (sample and sensor references) in the ForteBio's data analysis software. The analysis accounts for non-specific binding, background and signal drift and minimizes well based and sensor variability.

### Proteolytic fingerprinting

Proteolytic fingerprinting assay was conducted as described previously [38]. Briefly, Hsp90α (0.6 μg) was incubated with HDN-1 (0.2 mM) or 17-AAG (0.2 mM) in the assay buffer (10 mM Tris-HCl, 50 mM KCl, 5 mM MgCl_2_, 0.1 mM EDTA, pH 7.4) at 37°C for 5 min. The samples were digested on ice with TPCK-treated trypsin, which selectively cleaves peptide bonds C-terminal to lysine and arginine residues, for 6 min at the concentration of 30 and 150 μg/ml. The reactions were terminated by adding SDS sample buffer followed by boiling for 3–5 min. The digested products were analyzed by Western blotting with Hsp90 (AC88) antibody.

### Analysis of drug combination studies

H1975 cell were seeded in triplicate in 96-well plates and treated with HDN-1 or 17-AAG alone or with the combination of HDN-1 and 17-AAG at the indicated doses. MTT assays were performed after 72 h of treatment. The Q (value) was calculated according to Jin's formula: Q = E_a+b_/(E_a_+E_b_−E_a_ × E_b_). E_a+b_ represented the inhibition rate of drug combination, E_a_ and E_b_ represented the inhibition rate of drug A and B alone, respectively. Q (value) = 0.85–1.15 stands for additivity, Q (value) > 1.15–2.0 stands for synergism, and Q (value) < 0.85–0.55 stands for antagonism [18].

### Fluorescence polarization assays

Fluorescence polarization assays were performed under the following conditions: each 96-well plate contained 200 nM FITC-GA, Hsp90α (30 nM), and tested inhibitor (initial stock in DMSO) in a final volume of 100 μl. For each assay, background wells (buffer only), tracer controls (free, FITC-GA only), and bound GA controls (fluorescent GA in the presence of Hsp90) were included on each assay plate. The fraction of tracer bound to Hsp90 was correlated to the mP value and plotted against log10 values of competitor concentrations. The HDN-1 and 17-AAG concentrations at which 50% of bound GA was displaced was obtained by fitting the data. Fluorescence intensity measurements were obtained using a SpectraMax M5 (Molecular Devices, Sunnyvale, CA). The excitation and emission wavelengths were 485 nm and 530 nm, respectively.

### Molecular docking simulation

Three-dimensional structure building and all modeling were performed using the SYBYL-X 1.10 (Tripos Inc., St. Louis, MO, USA) Program Package [39] installed on a FOUNDER Wenxiang E520 workstation running the Red Hat Enterprise Linux (version 5) operating system. Energy minimization was performed using Powell optimization in the presence of the Tripos force field with a convergence criterion of 0.05 kcal/mol•Å and then assigned with the Gasteiger-Hückel charges. The 3D structures of Hsp90 (PDB: 2CGE) [40] was taken from the Research Collaboratory for Structural Bioinformatics Protein Data Bank (http://www.rcsb.org/pdb). Following the receptor's preparation, all hydrogen bonds were added and the side chain amides in all Asn and Gln oriented to maximize hydrogen bonding. Minimization was performed for 100 iterations using the AMBER FF99 force field [41] rflex-Dock Program interfaced with SYBYL-X was used to dock HDN-1 and chaetocin in the active sites of Hsp90.

### Stastical analysis

Data were presented as mean values ± standard deviation. Statistical comparisons among groups were performed by Student's *t*-test.
